# Authors' Reply: Teaching Health Workers Malaria Diagnosis

**DOI:** 10.1371/journal.pmed.0020165

**Published:** 2005-06-28

**Authors:** Graham Icke, Richard E Davis

**Affiliations:** **1**Royal Perth HospitalPerth, Western AustraliaAustralia

With reference to the letter commenting on our paper [1] from Drakeley and colleagues [2], they have some justification in stating that one size does not fit all. However our Web site, which is the basis for their comment, was originally designed on a wholly voluntary basis for Australia. We have been overwhelmed by the interest and acceptance of this site worldwide. Not only have we had more than 750,000 visitors to the site but have issued free CD-ROMs to institutions in 149 countries.

The fact that it was warmly embraced by so many others from around the world bears testimony to its usefulness as, indeed, do the tens of thousands of letters and E-mails thanking us for our efforts. We have made some modifications on our annual update in response to suggestions and changes in approach. The main section of interest has been the section on diagnosis, testing, and teaching, and perhaps the reason for this is the high quality of the illustrations. One only has to look at the site to recognize that we have not given equal weight to all sections, as Drakeley and colleagues point out. The emphasis is on diagnosis, testing, and teaching. Many large organizations have requested a substantial number of copies of the CD-ROM, and in Germany one organization has been printing its own (with permission). We are aware of some superb CD-ROMs on malaria put out from other sources, but they are expensive for organizations with a very small budget.

We accept that diagnosis by thick film is the norm in Africa and a number of other countries and, in fact, have spent many years ourselves diagnosing malaria from thick films in India and Southeast Asia. We have described how to make thick films and have provided a picture. We have mentioned the staining of films, but we have not described how to prepare the stains because we considered that outside our brief. It is important with Web sites to be concise, otherwise they won't be read. The actual diagnosis of malaria is the same for Africa, India, South America, and Southeast Asia, and it is the proper diagnosis that we believe is paramount. We know that language can be a serious problem. We have provided a version in French and Spanish. The French version we are told is useful for certain parts of Africa. When other languages have been requested, we have suggested that a small booklet should be written in the local language by those with local knowledge.

In regards to the comments on treatment, this section was written by T. M. E. Davis, who holds the Chair of Medicine at the University of Western Australia and is a consultant on malaria to Thailand and Cambodia. If we sought an expert on treatment for every endemic region, we would never get the material into print. One is not always able to use the drugs of choice in Southeast Asia because up to 50% of antimalarials sold in some areas are fake. It has been stated that children should not be given tetracyclines, but that has already been made very clear on the site.

We are aware that the site needs to take into account various interests and situations, which is why we have included our E-mail addresses on the site. We presume that as experts in the field Drakeley and colleagues would have been aware of the site either in Tanzania or the London School of Tropical Medicine, where a number of CD-ROMs have been requested and sent. We hope and expect that eventually a group with the enthusiasm of these correspondents will accept the challenge and produce a site that will overcome the problems that are the cause of their concern. We fully understand the difficulty of doing this on a voluntary basis. In the meantime, we will continue to service the site and hope that the very large number of users will continue to find it helpful. Finally, we state once again that if concise suggestions for improvements are sent to us by E-mail we will give them serious consideration. Our E-mail contact is now E-mail: sandy.treadgold@health.wa.gov.au.
